# Dissecting the roles of DR4, DR5 and c-FLIP in the regulation of Geranylgeranyltransferase I inhibition-mediated augmentation of TRAIL-induced apoptosis

**DOI:** 10.1186/1476-4598-9-23

**Published:** 2010-01-29

**Authors:** Shuzhen Chen, Lei Fu, Shruti M Raja, Ping Yue, Fadlo R Khuri, Shi-Yong Sun

**Affiliations:** 1 Department of Hematology and Medical Oncology, Emory University School of Medicine and Winship Cancer Institute, Atlanta, Georgia, USA

## Abstract

**Background:**

Geranylgeranyltransferase I (GGTase I) has emerged as a cancer therapeutic target. Accordingly, small molecules that inhibit GGTase I have been developed and exhibit encouraging anticancer activity in preclinical studies. However, their underlying anticancer mechanisms remain unclear. Here we have demonstrated a novel mechanism by which GGTase I inhibition modulates apoptosis.

**Results:**

The GGTase I inhibitor GGTI-298 induced apoptosis and augmented tumor necrosis factor-related apoptosis-inducing ligand (TRAIL)-induced apoptosis in human lung cancer cells. GGTI-298 induced DR4 and DR5 expression and reduced c-FLIP levels. Enforced c-FLIP expression or DR5 knockdown attenuated apoptosis induced by GGTI-298 and TRAIL combination. Surprisingly, DR4 knockdown sensitized cancer cells to GGTI298/TRAIL-induced apoptosis. The combination of GGTI-298 and TRAIL was more effective than each single agent in decreasing the levels of IκBα and p-Akt, implying that GGTI298/TRAIL activates NF-κB and inhibits Akt. Interestingly, knockdown of DR5, but not DR4, prevented GGTI298/TRAIL-induced IκBα and p-Akt reduction, suggesting that DR5 mediates reduction of IκBα and p-Akt induced by GGTI298/TRAIL. In contrast, DR4 knockdown further facilitated GGTI298/TRAIL-induced p-Akt reduction.

**Conclusions:**

Both DR5 induction and c-FLIP downregulation contribute to GGTI-298-mediated augmentation of TRAIL-induced apoptosis. Moreover, DR4 appears to play an opposite role to DR5 in regulation of GGTI/TRAIL-induced apoptotic signaling.

## Background

There are two major apoptotic signaling pathways: the intrinsic mitochondria-mediated pathway and the extrinsic death receptor-induced pathway, and these pathways are linked by the truncated proapoptotic protein Bid [1]. The extrinsic apoptotic pathway is negatively regulated primarily by the cellular FLICE-inhibitory protein (c-FLIP), including both long (FLIP_L_) and short (FLIP_S_) forms, through inhibition of caspase-8 activation, whereas the intrinsic apoptotic pathway is negatively regulated by multiple proteins including survivin [2]. The tumor necrosis factor-related apoptosis-inducing ligand (TRAIL) binds to its receptors: death receptor 4 (DR4, also named TRAIL-R1) and death receptor 5 (DR5, also named TRAIL-R2) to activate the extrinsic apoptotic pathway [3]. Recently TRAIL has received much attention because it preferentially induces apoptosis in transformed or malignant cells, demonstrating potential as a tumor-selective apoptosis-inducing cytokine for cancer therapy [3]. Currently TRAIL is being tested in phase I clinical trials. However, some cancer cells are resistant to TRAIL [4,5]. Thus agents that can sensitize cancer cells to TRAIL are useful to enhance the efficacy of TRAIL-based cancer therapy or to overcome TRAIL-resistance [6].

Protein geranylgeranyltransferase type I (GGTase I) is responsible for the posttranslational modification of CAAX motif-containing proteins such as K-Ras, N-Ras, RhoA, RhoC, Rac1, RalA, and Cdc42, which are often involved in cell transformation, tumor development and metastasis [7,8]. Thus, GGTase I has been considered a good cancer therapeutic target and thus great efforts have been made to develop GGTase I inhibitors as anticancer drugs [7,9]. Earlier studies using the GGTase I inhibitor, GGTI-287, showed K-Ras, a substrate for both GGTase I and farnesyltransferase (FTase), was particularly sensitive to GGTase I inhibition [10]. This finding was further supported by a genetic approach which has shown that GGTase I deficiency reduces tumor formation and improves survival in mice with K-Ras-induced lung cancer [11], thus further supporting the importance of GGTase I inhibition as a useful strategy to treat cancer, particularly K-Ras-induced cancer.

It has been documented that GGTase I inhibitors induce apoptosis in human cancer cells [12-16]. However, the underlying mechanisms are largely unknown although it appears to be associated with inhibition of Akt and downregulation of survivin and Mcl-1 [13,14]. The present study focused on examining the effects of the GGTase I inhibitor GGTI-298, as well as GGTI-DU40, on induction of apoptosis, particularly induced by TRAIL, in human non-small cell lung cancer (NSCLC) cells and on understanding underlying mechanisms.

## Methods

### Reagents

GGTI-298 was purchased from Sigma Chemical Co. (St. Louis, MO). GGTI-DU40 and its inactive analog SN-DU40 were described previously [17]. These agents were dissolved in DMSO at the concentration of 20 mM, and aliquots were stored at -80°C. Stock solutions were diluted to the appropriate concentrations with growth medium immediately before use. The soluble recombinant human TRAIL was purchased from PeproTech, Inc. (Rocky Hill, NJ). Rabbit polyclonal anti-DR5 antibody was purchased from ProSci Inc (Poway, CA). Mouse monoclonal anti-DR4 antibody (B-N28) was purchased from Diaclone (Stamford, CT). Mouse monoclonal anti-FLIP antibody (NF6) was purchased from Alexis Biochemicals (San Diego, CA). Mouse monoclonal anti-caspase-3 antibody was purchased from Imgenex (San Diego, CA). Rabbit polyclonal anti-caspase-8, anti-caspase-9, anti-PARP, anti-survivin and anti-phospho-Akt (p-Akt; Ser473) antibodies, and monoclonal anti-IκBα, and anti-phospho-IκBα (p-IκBα; Ser32/36) antibodies were purchased from Cell Signaling Technology, Inc. (Beverly, MA). Goat polyclonal anti-Rap1A (C-17; sc-1842) and rabbit polyclonal anti-RhoB (119) antibodies were purchased from Santa Cruz Biotechnology (Santa Cruz, CA). Rabbit polyclonal anti-β-actin and anti-tubulin antibodies were purchased from Sigma Chemical Co.

### Cell Lines and Cell Culture

Human NSCLC cell lines used in this study were purchased from the American Type Culture Collection (Manassas, VA). The stable transfectants H157-Lac Z-5, H157-FLIP_S_-1, A549-Lac Z-9 and A549-FLIP_S_-8 were described previously [18,19]. The NF-κB reporter stable cell line A549 with chromosomal integration of a luciferase reporter construct regulated by 6 copies of the NF-κB response element was purchased from Panomics, Inc (Fremont, CA). These cell lines were cultured in RPMI 1640 containing 5% fetal bovine serum at 37°C in a humidified atmosphere of 5% CO_2 _and 95% air.

### Cell Survival Assay

Cells were seeded in 96-well cell culture plates and treated the next day with the agents indicated. The viable cell number was determined using the sulforhodamine B assay, as previously described [20].

### Detection of Apoptosis

Apoptosis was evaluated by Annexin V staining using Annexin V-PE apoptosis detection kit purchased from BD Biosciences (San Jose, CA) following the manufacturer's instructions. Caspase activation was also detected by Western blotting (as described below) as an additional indicator of apoptosis.

### Western Blot Analysis

Whole-cell protein lysates were prepared and analyzed by Western blotting as described previously [21,22].

### Luciferase Activity Assay

The given cells were lysed with Reporter Lysis Buffer (Promega; Madison, WI) and subjected to luciferase activity assay using Luciferase Assay System (Promega) in a luminometer. Relative luciferase activity was normalized to protein content.

### Gene Silencing Using Small Interfering RNA (siRNA)

The non-silencing control, DR4 (#1), DR5 and c-FLIP siRNA duplexes were described previously [22-24]. Additional DR4 siRNAs that target different regions of DR4 gene were also used in this study. DR4 #2 and DR4 #3 siRNAs target 5'-AACGAGATTCTGAGCAACGCA-3' (954-974) and 5'-AATGAGATCGATGTGGTCAGA-3' (1239-1259), respectively. RhoB siRNA (sc-29475) was purchased from Santa Cruz Biotechnology. Transfection of these siRNA duplexes were conducted in 6-well or 96-well plates using the HiPerFect transfection reagent (Qiagen, Valencia CA) following the manufacturer's manual. Gene silencing effects, caspase activation and other protein expression were evaluated by Western blot analysis, whereas cell survival and apoptosis were measured by the SRB assay and Annexin V staining, respectively, as described above.

## Results

### GGTI-298 Induces Apoptosis of Human NSCLC Cells

**Figure 1:**
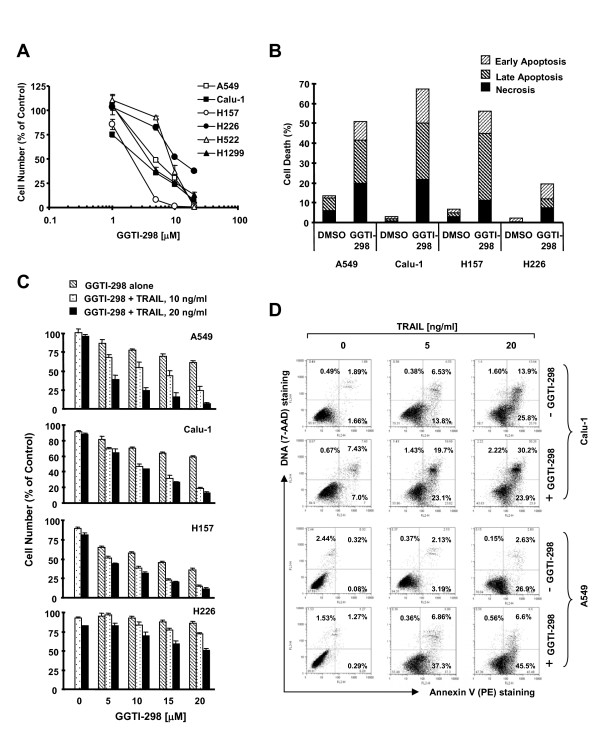
**GGTI-298 as a single agent (*A *and *B*) or combined with TRAIL (*C *and *D*) inhibits the growth (*A *and *C*) and induces apoptosis (*B *and *D*) of human NSCLC cells**. *A *and *C*, The indicted cell lines were seeded in 96-well cell culture plates and treated the next day with the given concentrations of GGTI-298 (*A*) or with the given concentrations of GGTI-298 alone, TRAIL alone, and their combinations as indicated (*C*). After 3 days (*A*) or 24 h (*C*), cell number was estimated using the SRB assay. Data are the means of four replicate determinations; Bars, ± SDs. *B *and *D*, The indicated cell lines were treated with 15 μM GGTI-298 for 48 h (*B*) or with the indicated doses of TRAIL alone, 15 μM GGTI-298 alone, and their respective combinations for 24 h (*D*). Apoptosis were then determined by Annexin V staining.

GGTI-298 was previously shown to induce G1 arrest and apoptosis in A549 lung cancer cells [15]. In this study, we compared the effects of GGTI-298 on cell growth and induction of apoptosis in a panel of human NSCLC cell lines. After a 3-day exposure, GGTI-298 exhibited concentration-dependent effects on decreasing the cell numbers of 6 NSCLC cell lines tested, with IC_50_s ranging between 2 to 10 μM, indicating that GGTI-298 effectively inhibits the growth of human NSCLC cells. Among these cell lines, the H226 cell line was the least sensitive to GGTI-298 (Fig. 1A). By Annexin V staining, we detected an increase in the number of apoptotic cells as well as necrotic cells in the four tested cell lines (i.e., A549, Calu-1, H157 and H226) exposed to GGTI-298 for 48 h, demonstrating that GGTI-298 induces cell death, particularly apoptotic cell death. Similarly, we detected the least apoptotic cells in H226 cells treated with GGTI-298, indicating that H226 cells were less sensitive to GGTI-298-induced apoptosis (Fig. 1B). In agreement with previous reports, we found that GGTI-298 also induced G1 arrest in these NSCLC cell lines (data not shown).

### GGTI-298 Cooperates with TRAIL to Augment Apoptosis in Human NSCLC Cells

GGTase I, like FTase, is a cytosolic heterodimer consisting of a specific β subunit and a common α subunit, which is used in FTase heterodimers as well [7]. We previously showed that FTase inhibitors cooperate with TRAIL to augment apoptosis [25,26]. The present study determined whether GGTI-298 possesses similar activity. To this end, we examined the effects of GGTI-298 combined with TRAIL on the induction of apoptosis in a panel of human NSCLC cells. As presented in Fig. 1C, the GGTI-298 and TRAIL combination was more potent than each single agent alone in decreasing the survival of the four NSCLC cell lines tested, A549, Calu-1, H157 and H226. Among these cell lines, H226 was the least sensitive cell line responding to the combination treatment. Moreover, we directly detected apoptosis in cells exposed to TRAIL in the absence and presence of GGTI-298. Compared with TRAIL or GGTI-298 alone, the combination of GGTI-298 and TRAIL induced more apoptosis in both Calu-1 and A549 cells (Fig. 1D). For example, in A549 cells, TRAIL alone at 5 ng/ml and 20 ng/ml caused approximately 5% and 30% apoptosis, respectively, and GGTI-298 alone at 15 μM induced only 2% apoptosis; however, the combination of GGTI-298 with 5 ng/ml or 20 ng/ml TRAIL induced more than 40% or 50% apoptosis, respectively (Fig. 1D). Collectively, these results indicate that GGTI-298 cooperates with TRAIL to augment induction of apoptosis in human NSCLC cells.

### GGTI-298 Modulates the Expression of DR4, DR5 and c-FLIP

**Figure 2:**
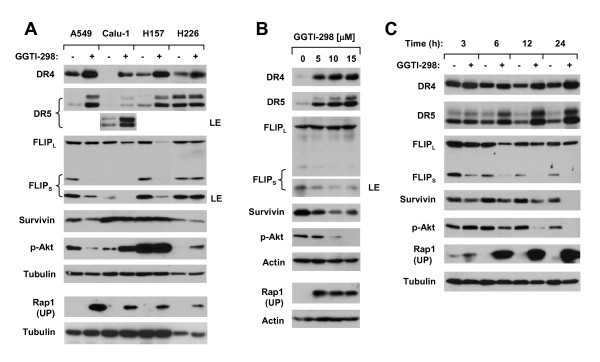
**GGTI-298 modulates the levels of DR4, DR5, c-FLIP, p-Akt and survivin in human NSCLC cells**. *A*, The given cell lines were treated with 15 μM GGTI-298 for 16 h. *B*, A549 cells were treated with the indicated concentrations of GTI-298 for 16 h. *C*, A549 cells were treated with 15 μM GGTI-298 for the given times. After the aforementioned treatments, the cells were subjected to preparation of whole-cell protein lysates and subsequent Western blotting. LE, longer exposure; UP, unprenylated.

Given that GGTI-298 cooperates with TRAIL to augment induction of apoptosis, we were interested in examining the underlying mechanisms. To this end, we determined whether GGTI-298 modulated the expression of DR4, DR5 and c-FLIP, which are key components in the TRAIL-mediated apoptotic pathway [27]. We found that GGTI-298 increased the expression of DR4 and DR5 while reducing the levels of c-FLIP, particularly the short form of c-FLIP (FLIP_S_) in A549, Calu-1 and H157 cells which are sensitive to GGTI-298 or the combination of GGTI-298 and TRAIL. However, GGTI-298 did not reduce c-FLIP levels or increased DR5 expression although it did increase DR4 expression in H226 cells which are less sensitive to undergo apoptosis by GGTI-298 or GGTI-298 plus TRAIL (Fig. 2A). In A549 cells, GGTI-298 modulated the levels of DR4, DR5 and FLIP_S _in both concentration- and time-dependent manners. GGTI-298 at even 5 μM increased expression of DR4 and DR5 and decreased FLIP_S _levels (Fig. 2B). Downregulation of FLIP_S _occurred at 3 h post GGTI-298 treatment; however, induction of DR4 or DR5 was detected after or at 6 h post GGTI-298 treatment (Fig. 2C), indicating that FLIP_S _downregulation occurs earlier than DR4 or DR5 induction in cells exposed to GGTI-298. We noted that FLIP_L _levels were reduced at early times (e.g., 3 h and 6 h) but not at late times (e.g., 12 and 24 h) (Fig. 2C). In H226 cells, GGTI-298 had minimal effects on upreuglating DR5 and reducing c-FLIP levels over the test time period from 3 h to 24 h (data not shown).

GGTI-298 was previously shown to inhibit Akt phosphorylation and downregulate survivin levels, which contribute to GGTI-induced apoptosis in ovarian cancer cells [14]. We also determined whether GGTI-298 exerted similar effects in human NSCLC cells. GGTI-298 indeed decreased the levels of p-Akt and survivin in A549 cells, which occurred even at 3 h post GGTI-298 treatment (Figs. 2A-C). These effects appeared cell line-dependent because GGTI-298 did not reduce the levels of p-Akt and survivin in the other three cell lines (i.e., Calu-1, H157 and H226 cells) (Fig. 2A). In fact, GGTI-298 even increased p-Akt levels in Calu-1 and H226 cells (Fig. 2A).

At the tested concentration ranges (5-15 μM), GGTI-298 inhibited the prenylation of Rap1 as indicated by an increase in the levels of unprenylated Rap1 (Fig. 2), a protein known to be modified by GGTase-1, indicating that GGTI-298 at the tested conditions inhibits protein geranylgeranylation.

### GGTI-298 Increases RhoB Expression, Which Partially Contributes to DR5 Induction

**Figure 3:**
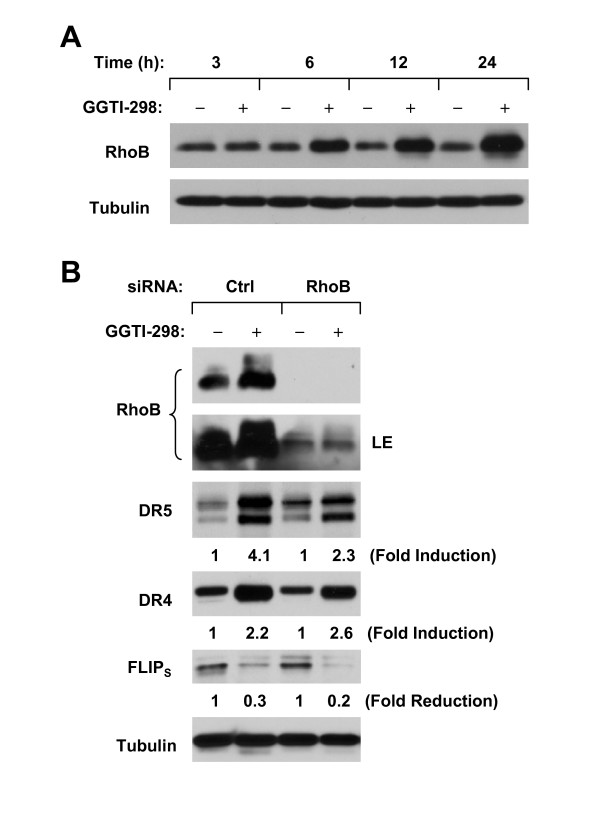
**RhoB is upregulationed by GGTI-298 (A) and in part mediates DR5 upregulation by GGTI-298 (B)**. *A*, A549 cells were treated with 15 μM GGTI-298 for the given times and then subjected to preparation of whole-cell protein lysates and subsequent Western blotting. *B*, A549 cells were cultured in a 6-well plate and the next day transfected with the indicated siRNAs. After 36 h, the cells were treated with 15 μM GGTI-298 for 12 h and then subjected to preparation of whole-cell protein lysates for Western blotting. Ctrl, control; LE, longer exposure.

It is known that RhoB is modified and upregulated by both FTase and GGTase inhibitors [28]. Given that both FTase and GGTase inhibitors induce DR5 expression based on our previous [25,26] and current studies (Fig. 2), we then determined whether RhoB modulation is involved in DR5 induction by GGTI-298. To this end, we first examine whether GGTI-298 increases RhoB expression in our cell systems. In agreement with previous findings [28], GGTI-298 strongly induced RhoB upregulation at 6 h post treatment (Fig. 3A). Transfection of RhoB siRNA abolished RhoB induction by GGTI-298. Subsequently, GGTI-298 increased DR5 levels by 4.1 fold in control siRNA-transfected cells, but only by 2.3 fold in RhoB siRNA-trasnfected cells (Fig. 3B), indicating that blockade of RhoB upregulation partially inhibits DR5 induction by GGTI-298. We also examined DR4 induction and FLIP_S _reduction in these cells and found that both DR4 induction and FLIP_S _decrease by GGTI-298 were basically same between control siRNA- and RhoB siRNA-trasnfected cells (Fig. 3B), indicating that Rhob upredulation is not responsible for GGTI-298-mediated DR4 induction and c-FLIP downregualtion.

### Another GGTase I Inhibitor GGTI-DU40 Exerts Similar Effects in Modulation of DR4, DR5 and c-FLIP Levels, and Augmentation of TRAIL-induced Apoptosis

**Figure 4:**
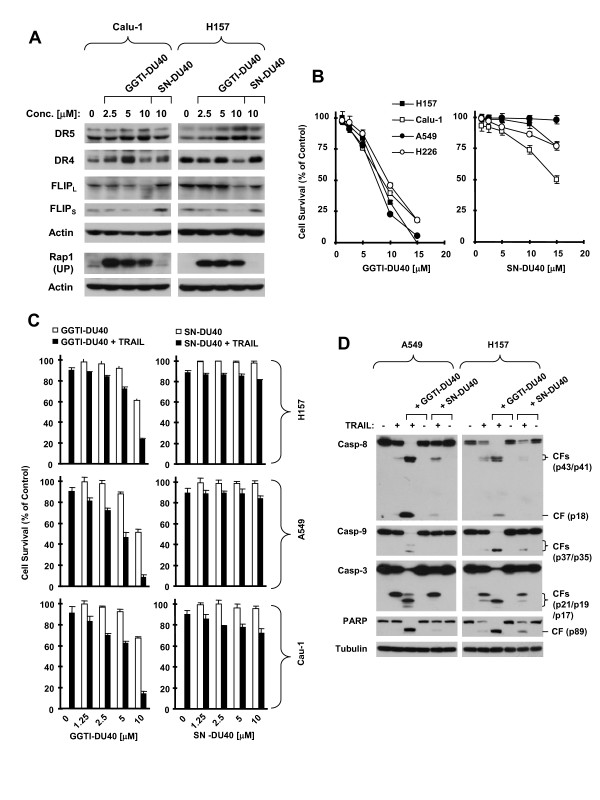
**Comparing the effects of GGTI-DU40 and SN-DU40 on modulation of DR5, DR4 and c-FLIP (*A*), on decreasing cell survival (*B*), and on TRAIL-induced apoptosis (*C *and *D*) in human NSCLC cells**. *A *and *D*, The indicated cell lines were treated with the given concentrations of GGTI-DU40 or SN-DU40 for 8 h (*A*) or with DMSO, 10 μM GGTI-DU40 or SN-DU40 alone, 20 ng/ml TRAIL alone of the combination of TRAIL with GGTI-DU40 or SN-DU40 for 6 h (D). The cells were then subjected to preparation of whole-cell protein lysates and subsequent Western blot analysis for the proteins as indicated. CF, cleaved form. *B *and *C*, The indicated cell lines were seeded in 96-well plates and treated with the given concentrations of GGTI-DU40 or SN-DU40 (*B*) or with 20 ng/ml TRAIL alone, the given concentrations of GGTI-DU40 or SN-DU40 alone, or the combination of GGTI-DU40 or SN-DU40 with TRAIL (*C*). After 3 days (*A*) or 24 h (*C*), the cells were subjected to the SRB assay for measurement of cell number. Data are means of four replicate determinations. Bars, ± SDs.

To determine whether other GGTase I inhibitors also modulate c-FLIP, DR4 and DR5 and augment TRAIL-induced apoptosis, we compared the effects of another highly selective GGTase I inhibitor, GGTI-DU40, and its structural analog, SN-DU40, which has minimal GGTase I-inhibitory activity [17], on modulation of c-FLIP, DR4, DR5 and TRAIL-induced apoptosis. Indeed, we confirmed that GGTI-DU40, but not SN-DU40, inhibited the prenylation of Rap1 in our cell systems (Fig. 4A). Compared to SN-DU40, which minimally or very weakly inhibited the growth of NSCLC cells, GGTI-DU40 effectively inhibited the growth of NSCLC cells with IC_50_s of around 10 μM or less (Fig. 4B). GGTI-DU40 at concentrations of 2.5-10 μM exerted concentration-dependent effects on downregulation of c-FLIP and induction of DR5 expression, whereas SN-DU40 at 10 μM only minimally or weakly modulated the levels of c-FLIP and DR5 in both Calu-1 and H157 cells (Fig. 4A). Consistently, GGTI-DU40, but not SN-DU40, enhanced TRAIL's effect on decreasing the survival of NSCLC cells in a concentration-dependent manner (Fig. 4C). In addition, GGTI-DU40, but not SN-DU40, when combined with TRAIL, exerted augmented effects on inducing cleavage of caspase-8, caspase-9, caspase-3 and PARP in both A549 and Calu-1 cells (Fig. 4D).

We noted that GGTI-DU40 at concentrations of 2.5 and 5 μM and SN-DU40 at 10 μM increased DR4 expression in Calu-1 cells; however they did not elevate DR4 levels in H157 cells. GGTI-DU40 at 10 μM even decreased the levels of DR4 in H157 cells (Fig. 4A). Nonetheless, these results may suggest that modulation of DR4 plays a less important role than the modulation of DR5 and c-FLIP in GGTase I inhibitor-mediated regulation of apoptosis.

### c-FLIP Downregulation is Important for Augmentation of TRAIL-induced Apoptosis by GGTI-298 and for GGTI-induced Apoptosis

**Figure 5:**
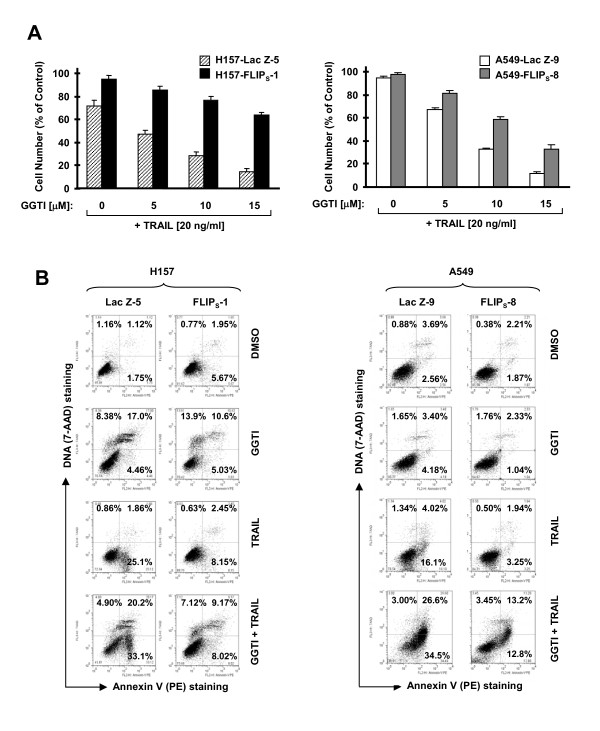
**Enforced expression of ectopic FLIP_S _attenuates the effects of the GGTI-298 and TRAIL combination on decreasing cell survival (*A*) and inducing apoptosis (*B*)**. *A*, The indicated transfectants were seeded in 96-well plates and treated with the indicated concentrations of GGTI-298 plus 20 ng/ml TRAIL. After 24 h, the cells were subjected to the SRB assay for measurement of cell number. Data are means of four replicate determinations. Bars, ± SDs. *B*, The indicated transfectants were treated with DMSO, 15 μM GGTI-298, 20 ng/ml TRAIL, and the combination of GGTI-298 and TRAIL for 24 h and then subjected to detection of apoptosis by Annexin V staining. GGTI, GGTI-298.

It is well known that c-FLIP is a major inhibitor of TRAIL-induced apoptosis [29]. Since GGTI-298 rapidly downregulates c-FLIP, particularly FLIP_S _levels, it is plausible to reason that FLIP_S _downregulation is critical for cooperative induction of apoptosis by GGTI-298 and TRAIL or for induction of apoptosis by GGTI-298. To test this hypothesis, we examined the impact of FLIP_S _overexpression on cell sensitivity to induction of apoptosis by GGTI-298 plus TRAIL. In both H157 and A549 cell lines, enforced expression of ectopic FLIP_S _conferred resistance to the combination of GGTI-298 and TRAIL both by measuring cell survival (Fig. 5A) and by detecting apoptotic cells with Annexin V staining (Fig. 5B). For example, GGTI-298 plus TRAIL induced 53% and 61% apoptosis, respectively, in H157-LacZ-5 and A549-Lac Z-9 control cell lines, but only 17% and 26% apoptosis, respectively, in H157-FLIP_S_-1 and A549-FLIP_S_-8 cells (Fig. 5B). Thus, enforced elevation of FLIP_S _levels protects cells from apoptosis induced by the GGTI-298 and TRAIL combination, suggesting that c-FLIP downregulation is critical for cooperative induction of apoptosis by the GGTI-298 and TRAIL combination.

**Figure 6:**
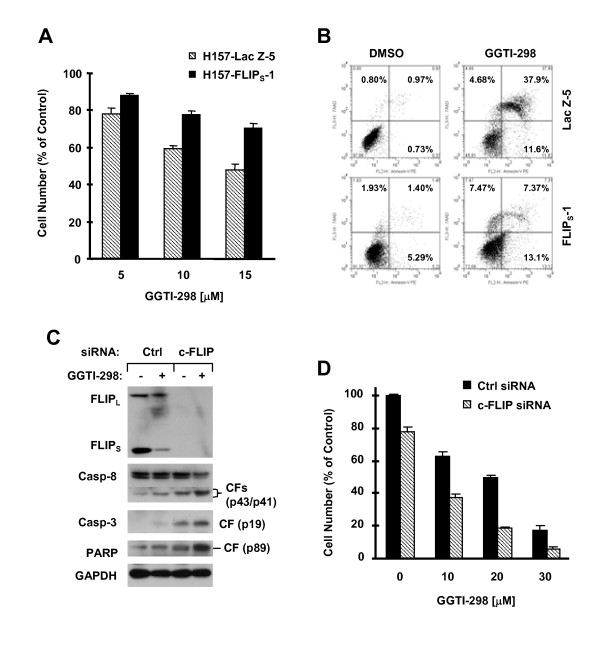
**Modulation of c-FLIP levels by enforcing expression of ectopic c-FLIP (*A *and *B*) or siRNA-mediated downregulation of c-FLIP (*C *and *D*) regulates cell sensitivity to GGTI-298-induced apoptosis**. *A *and *B*, The indicated transfectants were treated with the given concentrations of GGTI-298 (*A*) or 15 μM GGTI-298 (*B*) for 48 h and then subjected to estimation of cell number using the SRB assay (*A*) or detection of apoptosis by Annexin V staining (*B*). *C *and *D*, H226 cells seeded in a 6-well plate (*C*) or a 96-well (*D*) plate were transfected with control (Ctrl) or c-FLIP siRNA. Twenty-four hours later, the cells were exposed to 30 μM (*C*) or the indicated concentrations (*D*) of GGTI-298. After 24 h, the cells were subjected to preparation of whole-cell protein lysates and subsequent detection of the indicated proteins using Western blotting (*C*) or to estimation of cell number by the SRB assay (*D*). Data in *A *and *D *are means of four replicate determinations. Bars, ± SDs. CF, cleaved form.

Moreover, we determined whether enforced FLIP_S _expression made cells less sensitive to GGTI-298-induced apoptosis. As shown in Fig. 6, GGTI-298 decreased cell survival more in H157-LacZ-5 than in H157-FLIP_S_-1 cells (Fig. 6A). Consistently, GGTI-298 induced more apoptotic cells in H157-LacZ-5 (approximately 50%) than in H157-FLIP_S_-1 cells (approximately 20%) (Fig. 6B). These results indicate that enforced expression of FLIP_S _confers cell resistance to GGTI-298-induced apoptosis. H226 cells in which c-FLIP levels are not reduced by GGTI-298 are not sensitive to GGTI-induced apoptosis. We speculated that enforced downregulation of c-FLIP would sensitize the H266 cells to GGTI-298-induced apoptosis if c-FLIP levels determine cell sensitivity to GGTI-298-induced apoptosis. Thus, we reduced c-FLIP levels through siRNA-mediated gene silencing and then examined cell response to GGTI-298. As presented in Fig. 6C, transfection of c-FLIP siRNA substantially reduced the levels of c-FLIP (both FLIP_L _and FLIP_S_). GGTI-298 at high dose of 30 μM reduced the levels of both FLIP_L _and FLIP_S _with moderate effects on cleavage of caspase-8, caspase-3 and PARP in control siRNA-transfected cells. However, in c-FLIP siRNA-transfected cells, the effects of GGTI-298 on cleavage of caspase-8, caspase-3 and PARP were drastically enhanced when comparing the levels of the proforms and cleaved forms of these proteins. In agreement, GGTI-298 decreased cell numbers more effectively in c-FLIP siRNA-transfected cells than in control siRNA-transfected cells (Fig. 6D). For example, c-FLIP silencing alone caused about 20% cell number decrease and GGTI-298 alone at 10 μM and 20 μM decreased cell number by approximately 40% and 50%, respectively (in control siRNA-transfected cells). However, their respective combinations decreased cell number by more than 60% and 80%, respectively (in c-FLIP siRNA-transfected cells) (Fig. 6D), showing an additive or more than additive effect on decreasing cell survival. Taken data from Figs. 6C and 6D together, we suggest that enforced downregulation of c-FLIP levels sensitizes cells to GGTI-298-induced apoptosis. Collectively, we suggest that downregulation of c-FLIP is also important for GGTI-298-induced apoptosis.

### GGTI-298 Enhances TRAIL-induced Apoptosis Involving DR5 Upregulation

**Figure 7:**
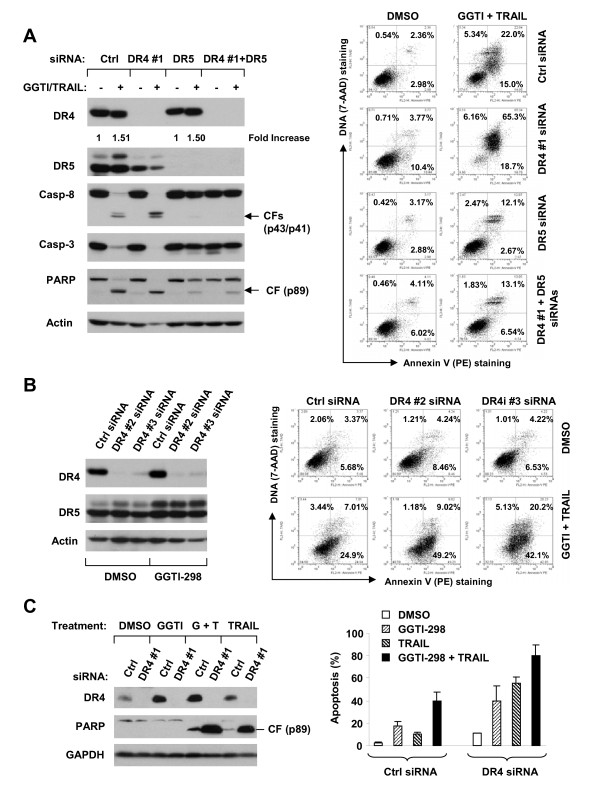
**Blockage of DR5 induction abrogates augmented induction of apoptosis by GGTI-298 and TRAIL combination (*A*), whereas inhibition of DR4 induction sensitizes cells to GGTI-298 plus TRAIL-induced apoptosis (*A *-C)**. *A *and *B*, A549 cells were cultured in a 6-well plate and the next day transfected with the indicated siRNAs twice in a 24 h interval. Twenty four hours after the second transfection, cells were treated with 15 μM GGTI-298 plus 20 ng/ml TRAIL (*A *and *B*, right panel) or 15 μM GGTI-298 only (*B*, left panel) for 8 h and then subjected to preparation of whole-cell protein lysates for Western blotting (*A *and *B*, left panels) or detection of apoptosis by Annexin V staining (*A *and *B*, right panels). *C*, H157 cells were cultured in a 6-well plate and the next day transfected with the indicated siRNAs twice in a 24 h interval. Twenty four hours after transfection, the cells were treated with 15 μM GGTI-298, 20 ng/ml TRAIL or GGTI-298 plus TRAIL. After 8 h (left panel) and 24 h (right panel), the cells were harvested for preparation of whole-cell protein lysates for Western blotting (left panel) or for detection of apoptosis by the Annexin V staining (right panel), respectively. Data are means of duplicate experiments; Bars, ± SEs. Ctrl, control; GGTI, GGTI-298; G + T, GGTI-298 plus TRAIL; CF, cleaved from.

Ligation of TRAIL with its death receptors including DR4 and DR5 is the first step in transducing apoptotic signaling. Thus, it is reasonable to speculate that upregulation of DR4 and DR5 also contributes to augmentation of TRAIL-induced apoptosis by GGTI-298. To prove this, we blocked induction of DR4, DR5 or both DR4 and DR5 though siRNA-mediated gene silencing and then determined the impact on GGTI-298 plus TRAIL-induced apoptosis. As presented in Fig. 7A (left panel), transfection of either DR4 or DR5 siRNA successfully decreased the basal levels and induced levels of DR4 or DR5 compared to transfection of the control siRNA. GGTI-298 plus TRAIL induced cleavage of caspase-8, caspase-3 and PARP in cells transfected with the control siRNA or DR4 siRNA, but only minimally in cells transfected with DR5 siRNA or both DR4 and DR5 siRNAs. Consistently, Annexin V staining also demonstrated that the GGTI-298 and TRAIL combination induced less apoptosis in cells transfected with DR5 siRNA (15%) or both DR4 and DR5 siRNAs (20%) than in cells transfected with the control (37%) or DR4 siRNA (84%) (Fig. 7A, right panel). We noted that DR4 silencing in fact made cells more sensitive to GGTI-298 plus TRAIL-induced apoptosis. Nonetheless, blockade of DR5 induction attenuates cooperative induction of apoptosis by the combination of GGTI-298 and TRAIL.

To further demonstrate the role of DR4 induction in GGTI-298 plus TRAIL-induced apoptosis, we used two additional DR4 siRNAs that target different regions of the DR4 gene. Similarly, both DR4 #2 and DR4 #3 siRNA effectively decreased basal levels of DR4 and blocked GGTI-298-induced DR4 expression without affecting DR5 expression (Fig. 7B, left panel). Under such conditions, GGTI-298 plus TRAIL induced 32% apoptosis in control siRNA-transfected cells, but 58% and 62% apoptosis, respectively, in DR4 #2 siRNA-transfected and DR4 #3 siRNA-transfected cells (Fig. 7B, right panel). These results again indicate that DR4 silencing sensitizes cells to induction of apoptosis by the combination of GGTI-298 and TRAIL.

The results on silencing of DR4 expression-mediated sensitization of A549 cells to GGTI-298 plus TRAIL-induced apoptosis appear surprising. We further reproduced this finding in the H157 NSCLC cell line, in which both DR4 and DR5 are induced by GGTI-298. In agreement with the results in A549 cells, blockade of GGTI-298-induced DR4 upregulation substantially enhanced GGTI-298 plus TRAIL-induced PARP cleavage and apoptosis (80% vs. 40% apoptosis in DR4 siRNA-transfected cells vs. control siRNA-transfected cells) (Fig. 7C). Therefore, the sensitization of GGTI-298 plus TRAIL-induced apoptosis by silencing DR4 is not restricted to only A549 cells.

### The Combination of GGTI-298 and TRAIL Facilitates IκBα Degradation and Akt Inhibition

**Figure 8:**
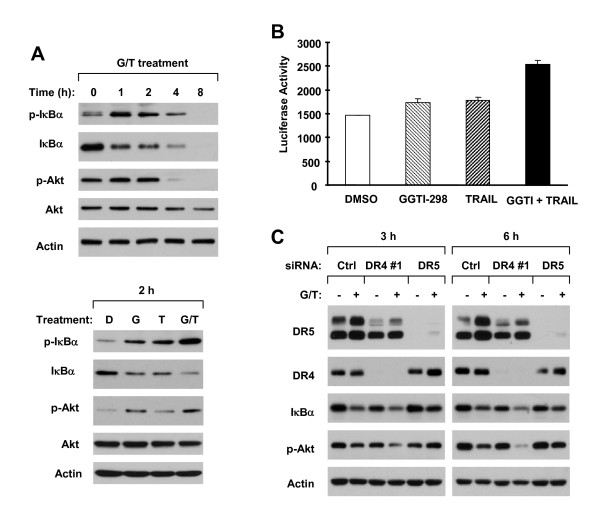
**The combination of GGTI-298 and TRAIL augments Akt inhibition, IκBα degradation (*A*) and NF-κB activation (*B*), which are differentially modulated by DR4 and DR5 (*C*)**. *A*, A549 cells were treated with the combination of 15 μM GGTI-298 and 20 ng/ml TRAIL for the given times (upper panel) or with DMSO (D), 15 μM GGTI-298 (G) alone, 20 ng/ml TRAIL (T) alone or GGTI-298 and TRAIL combination (G/T) for 2 h (lower panel). The cells were then subjected to preparation of whole-cell protein lysates and subsequent detection of the indicated proteins using Western blotting. *B*, A549 cells stably transfected with NF-κB-luc reporter gene were treated with DMSO, 15 μM GGTI-298 alone, 20 ng/ml TRAIL alone or GGTI-298 and TRAIL combination for 3 h. The cells were then lysed for NF-κB luciferase assay, which was finally normalized by protein content. The columns are means of triplicate determinations; Bars, ± SDs. *C*, A549 cells were cultured in 6-well plates and the next day transfected with the indicated siRNAs twice in a 48 h interval. Twenty-four hours after the second transfection, the cells were treated with 15 μM GGTI-298 plus 20 ng/ml TRAIL (G/T) for the indicated time and then subjected to preparation of whole-cell protein lysates and subsequent detection of the given proteins using Western blotting.

It has been shown that DR4 mediates NF-κB activation while inducing apoptosis although other studies showed that both DR4 and DR5 activate NF-κB [30,31]. Thus, it is possible that knockdown of DR4 abolishes NF-κB activation, leading to sensitization of DR5-dependent apoptosis induced by the combination of GGTI298 and TRAIL. To test this hypothesis, we first determined whether the GGTI-298 and TRAIL combination augments NF-κB activation by examining IκBα phosphorylation and degradation. The combination of GGTI-298 and TRAIL rapidly increased IκBα phosphorylation accompanied with reduction of IκBα levels at 1 h post the combination exposure (Fig. 8A, upper panel), indicating that the GGTI-298 and TRAIL combination induces IκBα degradation. The combination was more potent that either GGTI-298 or TRAIL alone in increasing p-IκBα levels and reducing IκBα levels (Fig. 8A, lower panel), indicating that the combination of GGTI-198 and TRAIL augments IκBα degradation. Given that IκBα degradation is often associated with NF-κB activation, we further determined whether the GGTI-298 and TRAIL combination enhances NF-κB activation. As shown in Fig. 8B, GGTI-298 and TRAIL alone weakly increased NF-κB activity as indicated by measuring NF-κB luciferase activity; however their combination caused the highest NF-κB activity. Thus, the GGTI-298 and TRAIL combination augments NF-κB activity as well.

Akt is another important survival pathway that maintain the growth of cancer cells by conferring apoptotic resistance [32]. Thus, we also looked at the effect of the GGTI-298 and TRAIL combination on Akt activation. The combination caused transient increase in p-Akt levels (e.g., 1 and 2 h) followed by substantial reduction of p-Akt levels (e.g., 4 h and 8 h) (Fig. 8A). These results indicate that prolonged exposure of cancer cells to the combination of GGTI-298 and TRAIL inhibits Akt phosphorylation.

### DR4 and DR5 Play Opposite Roles in Mediating GGTI-298 plus TRAIL-induced IκBα Degradation and Akt Inhibition

Moreover, we determined the roles of DR4 and DR5 in mediating GGTI-298 plus TRAIL-induced IκBα degradation and Akt inhibition To this end, we used siRNA to knock down DR4 or DR5 expression and then looked at their respective impact on IκBα degradation and Akt inhibition. Transfection of either DR4 or DR5 siRNA substantially reduced the levels of either DR4 or DR5 (Fig. 8C), indicating the successful knockdown of DR4 or DR5. Unexpectedly, knockdown of DR5, but not DR4, abrogated GGTI-298 plus TRAIL-induced reduction of both IκBα and p-Akt (Fig. 8C), indicating that the combination of GGTII-298 and TRAIL induces DR5-dependent IκBα degradation and Akt inhibition. Interestingly, silencing of DR4 even further enhanced the effects of the GGTI-298 and TRAIL combination on inhibition of Akt phosphorylation (Fig. 8C), suggesting that DR4 may play a role in preventing p-Akt downregulation during GGTI-298 plus TRAIL-induced apoptosis. Thus, it appears that DR4 and DR5 play opposite roles in mediating GGTI-298 plus TRAIL-induced Akt inhibition.

## Discussion

The present study demonstrates that GGTI-298 inhibits the growth of human NSCLC cells accompanied with induction of apoptosis in addition to G1 arrest, indicating that induction of apoptosis in part accounts for GGTI-298's growth inhibitory effects in human NSCLC cells. GGTI-298 as a single agent moderately induces apoptosis. However, GGTI-298 when combined with TRAIL exerted enhanced apoptosis-inducing activity, indicating that GGTI-298 cooperates with TRAIL to augment apoptosis. In agreement, another highly selective GGTase I inhibitor, GGTI-DU40, but not its inactive analog, SN-DU40, exerted similar effects in augmenting TRAIL-induced apoptosis. To the best of our knowledge, this is the first report demonstrating that inhibition of GGTase I cooperates with TRAIL to augment apoptosis in human cancer cells. Given the recent study that GGTase I deficiency reduces lung tumor formation [11], our data suggest that GGTase I inhibitors may be used in combination with TRAIL for treatment of human lung cancer through facilitating induction of apoptosis.

A previous report suggested that inhibition of Akt and subsequent downregulation of survivin are important for GGTI-298-induced apoptosis in human ovarian cancer cells [14]. In our study, we observed that GGTI-298 inhibited Akt phosphorylation and downregulated survivin levels primarily in A549 cells, but not in other tested NSCLC cell lines (e.g., Calu-1, H157 and H226), indicating a cell line-specific modulation of Akt and survivin. In Calu-1 cells, GGTI-298 even increased p-Akt levels. Since Calu-1 and H157 cells were also sensitive to induction of apoptosis by either GGTI-298 alone or GGTI-298 plus TRAIL, inhibition of Akt and survivin is unlikely to be a common mechanism for GGTI-298- or GGTI-298 plus TRAIL-induced apoptosis although they may play roles in individual cell lines (e.g., A549). Currently, we do not know why GGTI-298 decreases Akt phosphorylation in some cells lines (e.g., A549), but increases Akt phosphorylation in others (e.g., Calu-1). Similar phenomenon was also documented from studies on FTase inhibitors. Whereas FTase inhibitors decrease Akt phoshoporylation in some cells [33,34], we previously reported that the FTase inhibitor SCH66336 increases Akt phosphorylation in some human lung cancer cell lines [35].

Importantly, we found that GGTI-298 apparently downregulated the levels of c-FLIP, particularly FLIP_S _and induced the expression of DR4 and DR5, primarily in GGTI-sensitive cell lines (e.g., A549, Calu-1 and H157), suggesting an association between modulation of these proteins and cell sensitivity to GGTI-298 or GGTI-298 plus TRAIL. We noted that downregulation of c-FLIP preceded induction of DR4 and DR5, implying that c-FLIP downregulation may play a critical role in mediating GGTI-298-induced apoptosis and sensitization of TRAIL-induced apoptosis. Indeed, we found that enforced expression of ectopic FLIP_S _reduced cell sensitivity to GGTI-298-induced apoptosis, whereas siRNA-mediated silencing of c-FLIP further increased cell sensitivity to GGTI-298 (Fig. 6). Similarly, enforced expression of ectopic FLIP_S _also protected cells from GGTI-298 plus TRAIL-induced apoptosis (Fig. 5). Thus, we conclude that c-FLIP, particularly FLIP_S_, downregulation plays a crucial role in mediating apoptosis induced by either GGTI-298 or GGTI-298 combined with TRAIL.

Both DR4 and DR5 are receptors for TRAIL and can transduce apoptotic signaling upon binding to TRAIL. Interestingly, we found that silencing of DR5, but not DR4, abrogated cooperative induction of apoptosis by the combination of GGTI-298 and TRAIL (Fig. 7). Surprisingly, silencing of DR4 even sensitized cells to apoptosis induced by GGTI-298 plus TRAIL as we demonstrated using three DR4 siRNAs targeting different regions of the DR4 gene; however, this sensitization did not occur in cells where both DR4 and DR5 expression were silenced (Fig. 7). Taken together, these results indicate that DR5 induction is also critical for cooperative induction of apoptosis by the combination of GGTI-298 and TRAIL.

In this study, it appears that DR4 plays an antagonistic role in GGTI-298 plus TRAIL-induced apoptosis. Given that both DR4 and DR5 can mediate the TRAIL-induced NF-κB survival signaling pathway while initiating death signaling [30,31], it is possible that DR4 may be the major receptor that mediated TRAIL-induced NF-κB activation during TRAIL-induced apoptosis. Hence, silencing of DR4 would prevent NF-κB activation and enhance GGTI-298 plus TRAIL-induced apoptosis. Indeed, the GGTI-298 and TRAIL combination augmented IκBα phosphorylation, IκBα degradation and subsequent NF-κB activity (Fig. 8). Unexpectedly, knockdown of DR5, but not DR4, abrogated GGTI-298 plus TRAIL-induced IκBα degradation. Thus, the GGTI-298 and TRAIL combination induces a DR5-dependent IκBα degradation or NF-κB activation. Accordingly, we concluded that DR4 is unlikely to antagonize GGTI-298 plus TRAIL-induced apoptosis through activation of NF-κB.

Akt activation is often associated with apoptosis resistance [36]. In this study, we also found that the combination of GGTI-298 and TRAIL inhibited Akt phosphorylation. Interestingly, respective knockdown of DR4 and DR5 generated opposite results on GGTI-298 plus TRAIL-induced p-Akt reduction; i.e., knockdown of DR5 inhibited p-Akt reduction in cells exposed to the combination of GGTI-298 and TRAIL, whereas knockdown of DR4 enhanced the ability of the GGTI-298 and TRAIL combination to decrease p-Akt levels (Fig. 8C). Thus, the GGTI-298 and TRAIL combination induces a DR5-dependent Akt inhibition. Here it seems that DR4 may play an opposite role to DR5 in positively regulating Akt activity. Hence, it is possible that DR4 may mediate Akt activation, at least in part leading to cell resistance to GGTI-298 plus TRAIL-induced apoptosis. To the best of our knowledge, this is the first study to show the regulation of Akt activity by the TRAIL death receptors.

The finding on the opposite roles of DR4 and DR5 in regulation of GGTI-198/TRAIL-induced apoptosis is intriguing. However, the underlying molecular mechanism(s) have not been elucidated. It is possible that DR5 and DR4 can form a heterodimer, which is less active than a DR5 homodimer and even functions as an antagonist of DR5 homodimers. Knockdown of DR4 will favor DR5 homodimer formation while reducing the DR5/DR4 heterodimer formation, leading to sensitization of cells to GGTI-298/TRAIL-induced apoptosis. Nonetheless, our findings warrant further study in this direction.

Both GGTase I and FTase are cytosolic heterodimers that share a common α subunit but use different β subunits and are all involved in prenylation of CAAX proteins [7]. Our previous study showed that the FTase inhibitors SCH66336 and R115777 upregulate DR5 expression and augment TRAIL-induced apoptosis [25,26]. The current study further shows that the GGTase I inhibitor GGTI-298 exerts similar effects. Therefore, future studies should address whether there is a relationship between inhibition of protein prenylation and modulation of DR5 and c-FLIP. In this study, we have shown that GGTI-DU40, but not SN-DU40, exhibits identical results as GGTI-298 in modulation of c-FLIP levels, DR5 expression and TRAIL-induced apoptosis. Both GGTI-298 and GGTI-DU40 have similar GGTase I-inhibitory activity; however they possess distinct chemical structures [17,37]. All of these suggest a possible link between inhibition of protein geranylgeranylation and modulation of DR5 and c-FLIP.

Given that FTase and GGTase I inhibitors affect different set of proteins [8], the question is why both types of inhibitors exert similar effects on DR5 upregulation. One possible is that both FTase and GGTase I inhibitors affect a common protein or pathway, leading to DR5 upregulation. In this study, we investigated the possible role of RhoB in GGTI-298-induced DR5 upregulation since RhoB is modified and regulated by both farnesylation and geranylgeranylation [28]. We found that GGTI-298 increases RhoB expression (Fig. 3A), which was paralleled with DR5 induction (Fig. 2C). Moreover, siRNA-mediated blockade of RhoB upregulation partially inhibited DR5 induction by GGTI-298 (Fig. 3B). These results suggest that RhoB in part mediates DR5 induction by GGTI-298. Because RhoB siRNA transfection completely blocked RhoB upregulation in our study, but failed to abolish GGTI-298's ability to increase DR5 expression (Fig. 3B), other mechanisms may also account for GGTI-298-induced DR5 upregulation and need to be studied further.

## Conclusions

The current work focuses on addressing how inhibition of GGTase I cooperates with TRAIL to augment induction of apoptosis. In this regard, both c-FLIP downregualtion and DR5 induction are important events that mediate augmentation of TRAIL-induced apoptosis by GGTI-298. The unexpected finding on the antagonistic role of DR4 induction in regulation of GGTI-298/TRAIL-induced apoptosis is of interest and needs to be further explored.

## List of Abbreviations Used

DR: death receptor; GGTase: geranylgeranyltransferase; FLIP: cellular FLICE-inhibitory protein; FTase: farnesyltransferase; NSCLC: non-small cell lung cancer; siRNA: small interfering RNA; TRAIL: tumor necrosis factor-related apoptosis-inducing ligand.

## Competing interests

The authors declare that they have no competing interests.

## Authors' contributions

SC, LF, SMR and PY designed and conducted experiments as well data analysis. FRK participated in discussion of the data and draft of the manuscript. SYS participated in experimental design, coordination, data analysis and draft of the manuscript. All authors read and approved the final manuscript.
